# The FF domains of yeast U1 snRNP protein Prp40 mediate interactions with Luc7 and Snu71

**DOI:** 10.1186/1471-2091-9-29

**Published:** 2008-11-11

**Authors:** Claudia Ester, Peter Uetz

**Affiliations:** 1 Forschungszentrum Karlsruhe, Institute of Toxicology and Genetics, P. O. Box 3640, D-76021 Karlsruhe, Germany; 2 J Craig Venter Institute (JCVI), 9704 Medical Center Drive, Rockville, MD 20850, USA

## Abstract

**Background:**

The FF domain is conserved across all eukaryotes and usually acts as an adaptor module in RNA metabolism and transcription. *Saccharomyces cerevisiae *encodes two FF domain proteins, Prp40, a component of the U1 snRNP, and Ypr152c, a protein of unknown function. The structure of Prp40, its relationship to other proteins within the U1 snRNP, and its precise function remain little understood.

**Results:**

Here we have investigated the essentiality and interaction properties of the FF domains of yeast Prp40. We show that the C-terminal two FF domains of Prp40 are dispensable. Deletion of additional FF domains is lethal. The first FF domain of Prp40 binds to U1 protein Luc7 in yeast two-hybrid and GST pulldown experiments. FF domains 2 and 3 bind to Snu71, another known U1 protein. Peptide array screens identified binding sites for FF1-2 within Snu71 (NDVHY) and for FF1 within Luc7 (ϕ[FHL] × [KR] × [GHL] with ϕ being a hydrophobic amino acid).

**Conclusion:**

Prp40, Luc7, and Snu71 appear to form a subcomplex within the yeast U1snRNP. Our data suggests that the N-terminal FF domains are critical for these interactions. Crystallization of Prp40, Luc7, and Snu71 have failed so far but co-crystallization of pairs or the whole tri-complex may facilitate crystallographic and further functional analysis.

## Background

Spliceosome assembly in yeast occurs in a stepwise manner with U1, U2, U4/U6 and U5 snRNPs binding sequentially to the pre-mRNA and each other. The first defined step is the building of the commitment complex in yeast or the E complex in metazoans where the U1snRNP binds initially to the 5' splice site. The metazoan U1snRNP contains the proteins U1-A, U1-70K and U1-C. The yeast U1snRNP contains the homologs of these proteins, Mud1, Snp1 and Yhc1, as well as seven additional proteins [1-10].

In yeast the essential U1snRNP component Prp40 plays an important role in bringing the 5' and 3' splice sites into spatial proximity so that the intron can be spliced out of the pre-mRNA [1]. Ito et al[11] found Prp40 to interact with Snu71 among their "core" yeast two-hybrid (Y2H) data. To our knowledge, no direct interaction between Prp40 and Luc7 has been reported although both proteins have been found in the same complex multiple times (e.g. [12,13]).

**Figure 1:**
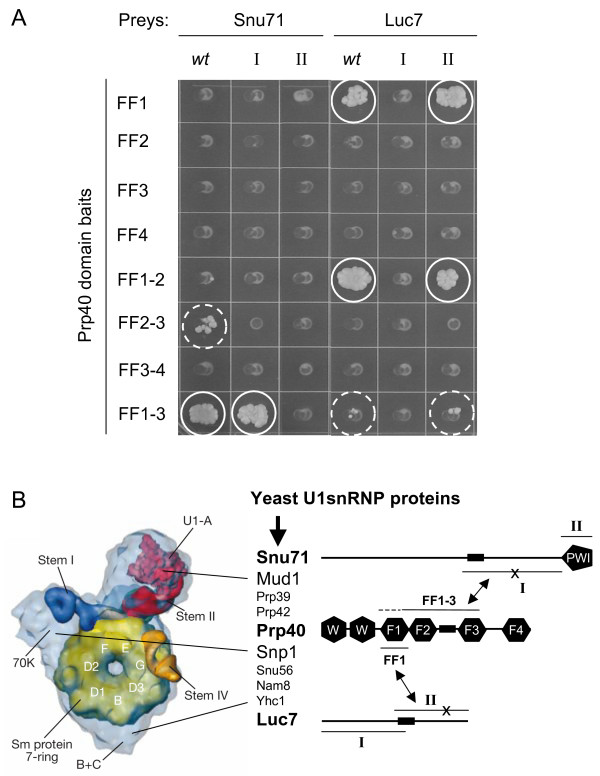
**Prp40 FF domains 1–3 bind to Snu71 I and FF domain 1 binds to Luc7 II**. Binding sites of FF domains and Luc7 and Snu71 were roughly mapped by Yeast-2-hybrid assays. **A**, Two-hybrid assay testing binding of Prp40 FF domain constructs (FF1, FF2, FF3; FF4, FF1-2, FF2-3, FF3-4, FF1-3) to full-length clones and fragments of Snu71 and Luc7 (for domains and fragments see panel B). Each encircled colony indicates a two-hybrid interaction (weak interactions in hatched circles). **B**, Schematic overview of Prp40 FF domain interactions (arrows) with the U1snRNP proteins Snu71 and Luc7 in the context of the U1snRNP as illustrated by the human U1snRNP (modified after [9]). Note that the structural model of the human U1snRNP does not include any of the proteins studied here. The dashed line extending to the FF1 domain indicates that the full length Snu71 interacts with FF2-3 whereas Snu71-I interacts only with domains FF1-3.

Prp40 is a modular protein consisting of a pair of WW domains followed by a series of four FF domains (Fig. 1). FF domains were first described in 1999 by Bedford & Leder as domains of about 60 amino acids including two conserved Phenylalanines (F) after which the domain was named [14]. Typically FF domains occur in tandem arrays. For example, among the 78 metazoan FF domain proteins listed in the SMART database [15], 13 have 2 or 3 FF domains, while all others have 4 to 6. The structure of three FF domains has been solved recently: the FF1 domain of the human protein HYPA/FBP11, the FF1 domain of the yeast protein Prp40, and the single FF domain of Urn1/Ypr152c [16-18]. FF domain proteins can be classified in two families: the p190 Rho GTPase-related proteins [19] and the WW/FF family whose members contain one or more WW domains followed by several FF domains. Three proteins of the latter family are known to recognize the phosphorylated C-terminal domain (CTD) of the RNA polymerase II via their FF domains, namely the human transcription elongation factor CA150, the human splicing factor FBP11 and the yeast splicing factor Prp40 [16,20-22]. Furthermore, FF domains of CA150 seem to use multiple independent binding sites rather than to bind cooperatively to proteins such as the transcription and splicing associated factor Tat-SF1 [23]. The FF1 domain of Prp40 is known to interact with Clf1 (Crooked neck-like factor), an essential and well conserved multifunctional protein [24]. The role of the second yeast FF protein, Urn1/Ypr152c, which contains one WW domain and one FF domain, may be a splicing factor too [18].

Here we investigated the role of the FF domains and their binding specificity. More specifically, our study aims to complement other data in order to define a consensus binding site for FF domains, such as those sites known for other domains such as the SH3 domain (whose consensus binding site is "proline-rich"). We addressed this problem by a combination of genome-wide yeast two-hybrid screens, in-vitro binding assays, peptide arrays, and genetic experiments.

We show that only two of the four FF domains of Prp40 are essential and explain this behaviour by their interactions with two other components of the yeast U1 snRNP. The binding site we found significantly differs from previously published binding sites of FF domains and thus implicates that FF domains are interaction modules with a wide range of specificities, in stark contrast to other domains such as SH3 or PDZ domains.

## Results

### Prp40 interacts with Luc7 and Snu71 via its FF domains

To investigate the interaction properties of yeast FF domains, we first screened Prp40 and Urn1/Ypr152c as well as several isolated FF domain baits against genome wide yeast two-hybrid arrays containing almost all ORFs of *Saccharomyces cerevisiae *as Gal4-activation domain fusions (preys [25]). These screens resulted in only two interaction partners, Snu71 and Luc7, two other known components of the U1 snRNP [12,13,26]. No interaction partners could be identified for the full-length Ypr152c protein, nor for its FF domain bait.

Once Prp40 was identified as interacting bait, we cloned its FF domains and combinations thereof and tested these as baits against fragments of Snu71 and Luc7 as preys (see methods for domain definitions). These experiments showed that the FF1 domain of Prp40 binds to a C-terminal fragment of Luc7 (Luc7 II, Fig. 1). Similarly, a C-terminal fragment of Snu71 (Snu71 I) binds to Prp40 FF domains 1–3. Interestingly, full length Snu71 but not Snu71-I interacts with the FF2-3 domain fragment (Fig. 1).

We have also screened isolated domains or combinations thereof as baits against our genome-wide prey array. However, no new proteins were found this way: FF1-4 interacted with Snu71 and Luc7, while FF1-3 and FF2-3 interacted only with Snu71 but not Luc7 in these screens (data not shown).

These results encouraged us to revisit the protein topology of the U1 snRNP. We tested all U1snRNP associated proteins by systematic pairwise yeast two-hybrid tests using full-length bait and prey constructs but found only the previously detected interactions Prp40-Snu71 and Prp40-Luc7.

**Figure 2:**
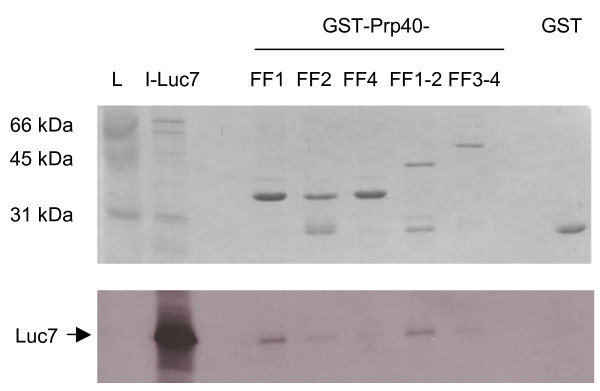
**The FF1 domain of Prp40 binds to Luc7 in vitro**. Various GST-Prp40FF domain fusion proteins (FF1, FF2; FF4, FF1-2, FF3-4) were tested for Luc7 binding *in vitro*. Top panel shows Coomassie blue-stained GST fusion proteins, while bottom row shows autoradiography of bound Luc7 protein. Luc7 bound to a construct containing FF1-2 domains as well as to the FF1 domain alone, but barely to FF2 alone. I, input; L, ladder (molecular mass marker).

To verify the yeast two-hybrid experiments by an independent method we performed GST pulldowns. Different FF domains were expressed as GST fusion proteins in *E. coli *and purified on glutathione sepharose beads. Surprisingly, protein constructs containing the FF3 domain consistently failed to be expressed in significant amounts. We conclude that the FF3 domain renders the FF3, FF2-3, FF1-3 and FF1-4 constructs somehow insoluble or unstable. Nevertheless, Snu71 and Luc7 were translated and 35S-labeled in vitro and incubated with the remaining GST-FF fusion proteins. These experiments clearly showed that Luc7 binds specifically to the FF1 and possibly FF2 domains of Prp40 although binding to FF2 was signficantly weaker than to FF1 (Fig. 2). Unfortunately, the interaction between Prp40-FF1-3 and Snu71 could not be confirmed this way as it involved the inaccessible FF3 domain.

### FF domains bind to specific peptides in Luc7 and Snu71

**Figure 3:**
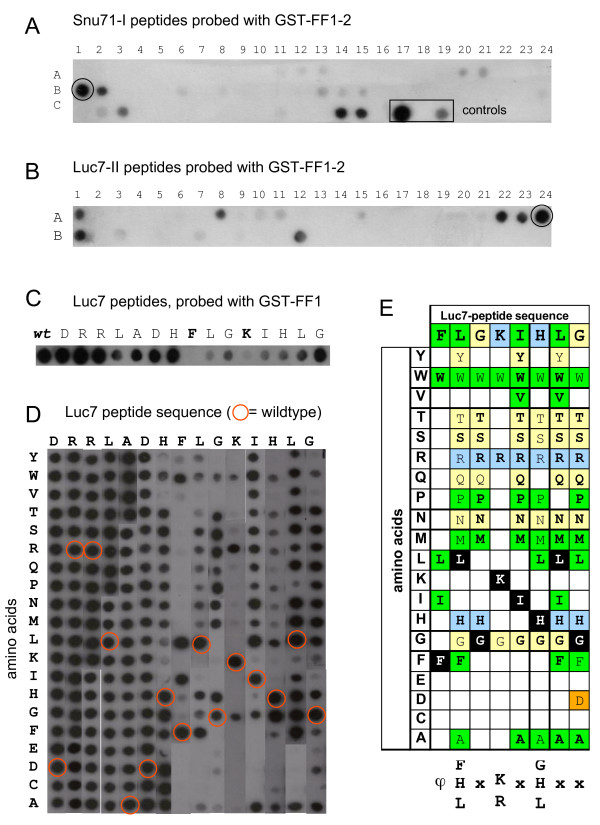
**FF domains of Prp40 bind to specific peptides within Snu71 and Luc7**. **A**, Peptides of Snu71-I (aa 329–536) and Luc7-II (aa 93–261) were synthesised as 15 mers with 3 aa shifts on a β-alanine membrane. A control peptide (QRALAKDLIVPRRP, position C17) binds to the α-GST antibody. Incubation with GST-FF1-2 showed a strong signal at peptide B1 and A24 (panel B). **B**, Peptide scan as in A but with Luc7 peptides probed with GST-FF1-2. Control membranes probed with GST alone were negative (data not shown). **C**, Alanine-scan of the peptide found in B (position A24, wildtype sequence: DRRLADHFLGKIHLG). Letters mark the amino acids which were replaced by alanine. GST-FF1 served as probe. The most important amino acid for the interaction are shown in bold. **D**, Substitution analysis of the Luc7 peptide sequence from C, probed with GST-FF1. Each residue within this sequence was substituted by 20 naturally occurring L-amino acids. All spots in circles represent the wild-type amino acids. All other spots are single substitution analogs, with rows defining the sequence position that is substituted and columns defining the amino acid that replaces the wild-type residue. **E**, The Luc7 motif bound by the FF1 domain of Prp40 as found in D. ϕ stands for hydrophobic amino acids, green indicates hydrophobic, yellow indicates polar, blue indicates basic and orange indicates acidic amino acids. White letters on black background represent the wildtype amino acids within the sequence.

The two-hybrid mapping experiments did not provide any information about the precise binding sites of the Prp40 FF domains within Luc7 and Snu71. In order to map the binding sites of the Prp40 FF domains we synthesized overlapping 15-mer peptides of Luc7 and Snu71 on nitrocellulose membranes and probed them with GST-FF domain fusions (Fig. 3). The peptides used covered the regions of Luc7 (amino acids 93–261) and Snu71 (amino acids 329–536) that were shown to interact in Y2H assays (Fig. 1). These screens identified a peptide in the C-terminal half of Luc7, D_214_RRLADHFLGKIHLG_228 _(A24), that appeared to be the primary interactor of the FF1 domain (Figs. 3B–D). Although the results shown in Figures 3A–B used a fusion of FF1-2 an explicit goal of this project was to identify targets of single FF domains. Hence the following experiments (Figs. 3C–D) were carried out with FF1 as this domain appeared to contribute most to binding (Fig. 2). The A24 peptide was characterized by systematic alanine scans (Fig. 3C) and substitution analysis (Fig. 3D) to define critical amino acids for the interaction with the FF1 domain of Prp40. This analysis clearly showed the importance of the FLGKIH motif for binding – only Glycine-223 and Histidine-226 seemed less important for binding. Based on our substitution analysis this interacting motif can be generalized as ϕ[FHL] × [KR] × [GHL] where ϕ may be any hydophobic amino acid (Fig. 3E). Interestingly, the Luc7 binding site showed no similarity to the interaction region (peptide B1: H_418_LANDVHYDHHRSFK_432_) in Snu71 which was obtained by the same approach (Fig. 3A). A consistent interaction region (N_421_DVHY) narrowed down by four overlapping peptides was detected when Snu71 II was synthesised on a CAPE-membrane as 20-mer overlapping peptides with 3 amino acid shifts and probed with GST-FF1-2.

### Only FF1 and FF2 domains are essential in yeast Prp40

**Figure 4:**
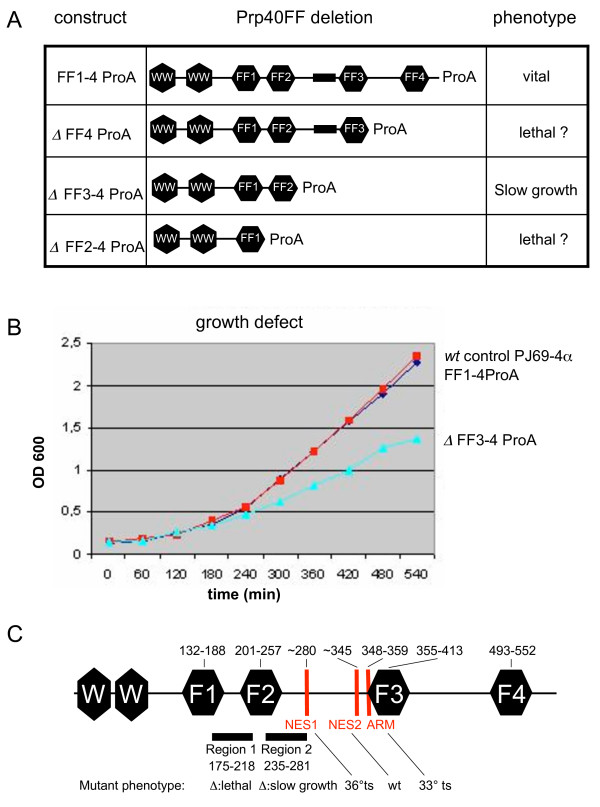
**Only Prp40 FF domains 1–2 are essential in vivo**. **A**, A strain with Protein A fused to the full-length Prp40 (FF1-4ProA) shows no signficant difference in growth to the wild-type. FF domain deletions from the C-terminus by insertion of Protein A into the respective FF domain locus resulted in a viable mutant that lacks FF domains 3–4. However, replacements of FF domains 4 or 3–4 could not be found and thus may be lethal. **B**, The mutant lacking FF domains 3–4 is viable but shows a growth defect compared to the wild-type controls. **C**, Summary of domains and functional motifs in Prp40. Numbers are amino acid sequence positions. Regions 1 and 2 are similar to RNA-binding domains. These and three other motifs, NES 1 and 2 (nuclear export signals) and an arginine-rich motif (ARM) have been deleted [Δ] or mutated by Murphy et al. [31]. The phenotypes of these mutations are shown below (ts = temperature-sensitive). Note that the predicted coiled-coil region indicated as bar between FF2 and FF3 in A is not shown here as it has no known function.

Although the two-hybrid mapping experiments indicated that the first two FF domains of Prp40 are the most important ones, their physiological role remained unclear. To determine the physiological role of the four FF domains in Prp40 we deleted one or more FF domains starting from the C-terminus in vivo (Fig. 4). In each mutant strain the deleted FF domain was replaced by a proteinA/kanMX6 cassette for selection of deletion mutants and easy protein detection (Fig. 4A). We were unable to isolate a deletion of FF domain 4 and thus assumed it to be essential. However, the deletion of FF domains 3–4 (FFΔ3-4ProA) was viable albeit it showed a clear growth defect when compared to two control constructs (FF1-4ProA, i.e. wildtype Prp40 with Protein A fused to its C-terminus and the wildtype control strain PJ69-4α) (Fig. 4B). Deletions of FF domains 2–4 and 1–4 appear to be lethal too as we could not isolate such mutants. These results confirmed our initial two-hybrid findings which indicated that FF1 and FF2 are critical for Prp40 whereas FF3 and FF4 may only increase its activity and/or stability.

### FFΔ3–4 deletions do not inhibit splicing of DBP2 and ECM33

Because Prp40 is a well-established component of the U1 snRNP, we reasoned that its phenotype is probably due to a defect in splicing. To investigate this question, we tested splicing efficiency in two selected *Saccharomyces cerevisiae *intron-containing genes, DBP2 and ECM33, which are commonly used as splicing reporters. However, RT-PCR assays of the RNAs of these two genes revealed no splicing defect in the FFΔ3–4 mutant (data not shown).

## Discussion

Although the composition of the the yeast U1 snRNP has been known for a decade [12,13] the precise interactions among its components and their atomic structure remain elusive. Equally puzzling is the fact that the human U1 snRNP is commonly assumed to consist of 10 proteins (U1-A, U1-70K and U1-C plus seven Lsm proteins (in addition to the snRNAs) [9,27] while its yeast counterpart contains up to 18 proteins [12,13]. In both species U1-A, U1-70K and U1-C (Mud1, Snp1, Yhc1 in yeast) form a complex that is associated with the heptameric Sm protein ring complex [9]. Except for the Mud1-Snp1-Yhc1 core complex and the Lsm ring little information is available about the structure of the yeast U1 snRNP. Most complex purification studies find large complexes of 10 to 51 proteins when individual U1 components are used as baits [10,28]. Here we suggest that Prp40, Luc7, and Snu71 form a subcomplex within the U1 snRNP. Surprisingly, we did not find any other interaction within the U1snRNP when we systematically tested all pairwise interactions among the known 10 subunits, suggesting that limitations of the yeast two-hybrid system or the requirement for RNA prevented detection of other interactions. Many of the U1snRNP associated proteins contain RNA binding domains and are known to bind RNA directly [29,30]. Interestingly, we did not find another known Prp40 interactor, Clf1 [24], in our initial genome-wide yeast two-hybrid screen. However, subsequent verification of the pertinent array position revealed that the Clf1 ORF was missing from our prey array.

We showed that the FF1 domain of Prp40 is responsible for the binding of Luc7 whereas the region FF1-3 binds to full length Snu71 as well as the C-terminal fragment Snu71-I. The fragment containing FF2-3 binds to full-length Snu71 but not to Snu71-I. This suggests that binding may be cooperative or that regions outside Snu71-I present additional binding sites. However, the single binding site indicates that there is no other strong site besides the N_421_DVHY motif. In any case, our results confirm that different FF domains clearly have different binding specificities with the FF1-2 region being the business end of Prp40 [17].

The ability of FF domains to bind non-phosphorylated peptides refutes the suggestion that the FF domain is an exclusively phospho-peptide binding domain.

The two FF domain binding sites identified by our study in Luc 7 and Snu71 do not share any obvious similarity with previously identified binding motifs (Table 1). Several studies showed that FF domains bind to phospho-peptides, usually the phospho-CTD of RNA polymerase II (Table 1). Similarly, several FF domains of CA150 were shown to bind to acidic peptides. However, while this may be true for Prp40 FF domains 2–3 the FF1 domain of this protein appears to prefer basic residues. This finding is in agreement with the acidic nature of Prp40-FF1 (binding basic peptides) and the basic FF2 and FF3 domains which bind the rather acidic Snu71 peptide. Unfortunately there is still too little information to derive reliable consensus binding sites. Similarly, available 3D structures of FF domains bound with their cognant ligands are not sufficient to derive rules that allow us to predict binding sites more generally. Clearly, more structural work is required to understand the binding mode of FF domains.

**Table 1:** Binding sites of FF domains

**FF protein**	**FF domain**	**Target protein**	**Binding site**	**Ref**.
Prp40	FF1	Clf1	GSTNIDILDLEELREYQRRKRTEYEGYLKRNRLD	[17]
Prp40	WW+FF1-2	RNAPII-CTD	YpSPTpSPS	[22]***
Prp40	FF1	Luc7	φ[FHL] × [KR] × [GHL] = FLGKIHLG	This study
Prp40	FF2-3	Snu71	NDVHY	This study
CA150	FF1,2,3**	Tat-SF1	(D/E)_2/5_-F/W/Y-(D/E)_2/5 _*	[23,21]
CA150	FF5	RNAP-CTD	YpSPTpSPS	[20]
FBP11	FF1	RNAP-CTD	YpSPTpSPS	[16]
Rho-GAP	FF1-4	TFII-I	(N-terminal 90 amino acids)	[15]

* i.e., two of the five residues either preceding or following an aromatic residue are negatively charged glutamic acid or asparagine.

** each of the FF1, FF2, and FF3 bound with similar affinity.

*** this interaction required both WW and FF domains. Neither of them was able to bind to the CTD by itself (pS indicates phospho-Serine).

Murphy et al. [31] have investigated the functional role of several motifs in Prp40, including putative RNA-binding domains they call "region 1" and "region 2" both of which overlap with the first two FF domains (Figure 4C). While deletion of region 1 is lethal, deletion of region 2 resulted in a slow growth phenotype. However, Murphy et al. were not able to show RNA-binding of these "domains" and thus it is likely that their similarity to RNA-binding domains is spurious. The fact that they overlap significantly with the FF domains supports that notion. Nevertheless, deletion of region 2 partially deletes the FF2 domain. It is possible that such a truncated FF2 domains has some residual binding activity and thus shows only a "slow growth" defect. We have not tested whether this partial deletion of FF2 still binds to Snu71 but given the non-essential role of Snu71, binding may not be absolutely required for U1 function.

The role of Snu71 remains elusive too. Newo et al. [32] have analyzed the U1 snRNP of *Schizosaccharomyces pombe *and found no homolog of Snu71 although homologs of Prp40 and Luc7 were clearly present. However, they speculate that Usp107p may be a functional homolog of Snu71, given its similar size and the presence of a PWI domain in Usp107p.

While Prp40 does not bind to a conserved sequence in Snu71, the binding site in Luc7 is highly conserved. As a more rigorous confirmation of the specificity of the Prp40 and other FF domains, it would be interesting to compare the peptide-binding specificities of all available domains with their cognate peptides under comparable conditions. Similarly, the *in vivo *relevance of the interactions described here can only be elucidated with detailed structural analysis and mutation of the interaction epitopes *in vivo*.

For a detailed understanding of U1 protein function, crystal structures of the individual proteins or, preferably, the whole complex, will be required. The structure would also tell us whether the FF domains of Prp40 bind to 3-dimensional epitopes or two linear motifs. Since membrane-bound peptides as used in this study may not be folded as in the native protein, they may produce artifactual results.

This study should also provide new insight into the important role of Prp40 as a mediator between transcription and splicing. While Prp40 has been consistently shown to be a component of the U1 snRNP, its precise role in splicing remains unclear. Similarly, the mechanistic details of its involvement in transcription require additional data. Several publications indicate a direct connection between specific steps of transcription and mRNA-processing in eukaryotes, i. e. co-transcriptional mRNA-processing [33-35].

## Conclusion

Our results show that FF domains 1 and 2 are critical for Prp40 function. However, while FF domains 3 and 4 are dispensable, they convey a considerable growth disadvantage when absent. We conclude that they also assist with spliceosome assembly or activity. This is also reflected by the evolutionary conservation of 4 or, sometimes 5, FF domains in homologous proteins. We suggest that our observations may also help to characterize the U1 snRNP structurally and suggest that previous crystallization efforts failed because Luc7 and Snu71 were expressed individually and crystallization attempts included only such individual proteins. We speculate that Luc7 may be crystallized together with Prp40 or fragments thereof. Further studies are required to derive general rules for physiological FF domain functions and activities.

## Methods

### 2.1. Plasmids and Strains

pOBD2 and pOAD [36] low-copy plasmids were used to express fusions of the Gal4-Gal4 activation domain (AD) (preys) and Gal4 DNA binding domain (DBD) (baits).

Construction of the Gal4_DBD_-ORF fusions was performed by means of PCR and recombination [37] as described in [25]. Transformation was performed using the lithium acetate procedure [38]. Bait constructs were transformed into yeast strain PJ69-4α [36] and prey constructs into PJ69-4a [39].

For Y2H mapping experiments FF domain constructs from Prp40 as baits and from Snu71 as prey were produced using the following primers (forward primers shown as codons): ForwardFF1: A TTC CAG CTG ACC ACC ATG AGA AGG ACT AAA GAA GAA, ReverseFF1: GA TCC CCG GGA ATT GCC ATG TGT TTC ATT GTG TTC CT, ForwardFF2: A TTC CAG CTG ACC ACC ATG AAG GAA CAC AAT GAA ACA, ReverseFF2: GAT CCC CGG GAA TTG CCA TGG ATT CTT TCT GAG TGT CG, ForwardFF3: A TTC CAG CTG ACC ACC ATG AAT TAT ACC AGA GAC CGT, ReverseFF3: ATC CCC GGG AAT TGC CAT GAC GTC TGT TGG GCT ATT G, ForwardFF4: A TTC CAG CTG ACC ACC ATG CAA AAT GAG CGT AGG ATA, ReverseFF4: GAT CCC CGG GAA TTG CCA TGC GCT TTC GGC AGT CGG, ForwardSnu71II: AA TTC CAG CTG ACC ACC ATG TCC GAG AGA AGC GCG GCA GAG, ReverseSnu71II: GAT CCC CGG GAA TTG CCA TGC TCT GCC GCG CTT CTC TCG GA, ForwardSnu71I: AA TTC CAG CTG ACC ACC ATG GCC AAA GGG AGC GCC AAT ACA, ReverseSnu71I: GAT CCC CGG GAA TTG CCA TGT GTA TTG GCG CTC CCT TTG GC. PCR reactions using these primers resulted in the following Gal4-DBD and/or GST fusion constructs (all based on wildtype sequences from ): amino acids 1-583 (Prp40wt), 1-75 (Prp40WW1-2); 129–560 (Prp40FF1-4); 129–428 (Prp40FF1-3); 129–264 (Prp40FF1-2); 196–428 (Prp40FF2-3); 351–560 (Prp40FF3-4); 129–201 (Prp40FF1); 196–264 (Prp40FF2); 351–428 (Prp40FF3); 487–560 (Prp40FF4); 1–620 (Snu71 wt); 530–620 (Snu71I); 329–536 (Snu71 II).

Luc7 constructs were created using existing restriction sites within its open reading frame (Luc7I: *StuI*, DNA position429; Luc7II, *EcoRI *DNA position 274).

Constructs expressing the Gal4_DBD_- and Gal4_AD_-ORF fusions were verified by DNA sequencing.

### 2.2. Two-Hybrid Screens, Retests and Mapping Experiments

An array containing most of the ~6,000 *Saccharomyces cerevisiae *ORFs expressed as Gal4_AD _fusions was used to screen for interacting proteins. Haploid transformants expressing either a full-length Gal4_DBD_-ORF fusion protein or a Gal4_DBD_-FF domain construct fusion protein were mated to the array [36]. The resulting diploids were pinned with a Biomek 2000 Laboratory Automation Workstation (Beckman-Coulter, Fullerton, CA) onto selective media as described in detail in [36]. Positive prey clones from a first-round screen were re-arrayed and also tested in single tests for reproducibility. Deletion constructs for the mapping yeast two-hybrid tests were obtained either by PCR or by digestion with compatible restriction enzymes (see section 2.1). These constructs were then tested as preys with the existing Prp40 FF bait constructs using standard Y2H protocols as they were used for the genome-wide screens.

### 2.3. Protein Expression

GST and GST-Prp40 constructs were expressed in *Escherichia coli *BL21 and purified using glutathione Sepharose 4B beads (Amersham Pharmacia, Uppsala, Sweden) as described in [40].

### 2.4. GST pull down assays

Modified primers for Luc7 and Snu71, containing a T7 promotor and a eukaryotic translation initiation site were used to generate PCR products for use with the T_N_T™-coupled reticulocyte system in the presence of [^35^S] methionine (Promega, Madison, WI). PCR primers were as follows: Luc7p *Forward*: GGATCCTAATACGACTCACTATAG GGAAACAGCCACCATGTCAACTATGTCAACGCCT, Luc7p *Reverse*: CTA CAC AAA GCG TCT TCC GGG; Snu71p *Forward: *ATCCTAATACGACTCACTATAG GGAAACAGCCACCATGAGGGAT ATTGTATTTGTA, Snu71p *Reverse*: TCA GGT CCC CAA GCG AAA TTC.

GST-FF domain fusion proteins or GST alone were coupled to glutathione-Sepharose beads (Amersham Pharmacia Biotech) and incubated with 4 μl of in vitro-translated proteins in pulldown buffer (40 mM Hepes pH 8.0; 2.5 mM MgCl_2_; 0,1 mM EDTA; 1 mM DTT; 1 mM PMSF; 0,2% Triton-X-100; 100 mM NaCl) for 2 h at 4°C under rotation. Beads with bound proteins were washed six times (for 10 min under rotation at 4°C) with pulldown buffer and proteins harvested in SDS-sample buffer, separated by SDS-PAGE, and analyzed by autoradiography.

### 2.5. Peptide SPOT synthesis

Cellulose membrane-bound peptides were prepared according to standard SPOT synthesis protocols [41,42] using an automated Spot synthesizer (MultiPep, Intavis AG Bioanalytical Instruments, Köln, Germany); Fmoc derivatives of amino-acids for peptide synthesis were obtained from Novabiochem. The generated peptide arrays were synthesized on amino-PEG membranes (Intavis AG Bioanalytical Instruments, Köln, Germany) or β-alanine membranes. β-alanine membranes were produced using hardened low ash whatman 50 paper incubated over night with Fmoc-β-alanine. After Fmoc-deprotection membranes were spotted with Fmoc-β-alanine-Opfp (Bachem AG, Switzerland) as spacer.

### 2.6. SPOT Membrane Probing

After activation of the membranes with methanol the membrane-bound peptide arrays were blocked 3 h in blocking buffer (2% milk powder and 5% sucrose in Tris-buffered saline (TBS), pH 8.0) and then incubated overnight at 4°C with 10 μg/ml purified GST fusion protein or GST control protein in blocking buffer. After washing three times with TBS the membranes were probed with anti-GST antibody (G1160; Sigma-Aldrich, München, Germany) in blocking buffer with 0.2% Tween for 3 h. The membrane was washed three times with TBS and then incubated for 1.5 h with horseradish-peroxidase-coupled anti-mouse mAb (Sigma-Aldrich; München, Germany) in blocking buffer with 0.2% Tween followed by washing three times with TBS. Analysis and quantification of peptide-bound GST fusion proteins were carried out using ECL (Amersham Biosciences, Freiburg, Germany). QRALAKDLIVPRRP is known to bind GST and was used as a positive control.

### 2.7. In vivo FF-domain Deletion

In order to delete the FF domain *in vivo *PCR products were created containing a protein A/*kanMX6 *cassette from the pYM8-plasmid [43] which were recombined into the Prp40 locus where they replaced the endogenous FF domains as shown in Fig. 4A. PCR-constructs were transformed into the PJ69-4α strain and plated on geniticin plates [200 mg/l] for selection. Genomic DNA was prepared from geniticin positive colonies and the correct deletion verified by PCR. PCR-products for recombination were prepared using the following primers: **Prp40ProA: ***Forward*, GCG TCA AAA AAG AGG CAT TTA ACT CCG GCT GTG GAA TTG GAC TAT CGT ACG CTG CAG GTC GAC, *Reverse*, ATA ATT TAT ATA ATG ATT AAC AAG ATA GAG GTC GAC ACG TCA GAA ATC GAT GAA TTC GAG CTC G; **ΔFF4ProA: ***Forward*, AGA AAC GAA AAG ATA CAA CAG AAA CTC CAA AAT GAG CGT AGG ATA CGT ACG CTG CAG GTC GAC *Reverse*, ATA ATT TAT ATA ATG ATT AAC AAG ATA GAG GTC GAC ACG TCA GAA ATC GAT GAA TTC GAG CTC G; **ΔFF3-4ProA: ***Forward*, CTT CAA AAC AAA CTA AAT GAG CTC CGA CTG CGC AAT TAT ACC AGA CGT ACG CTG CAG GTC GAC; *Reverse*, ATA ATT TAT ATA ATG ATT AAC AAG ATA GAG GTC GAC ACG TCA GAA ATC GAT GAA TTC GAG CTC G; **ΔFF2-4ProA: ***Forward*, CTT TCC AAT AGA TCA GCC GAT CAA CTT CTT AAG GAA CAC AAT GAA CGT ACG CTG CAG GTC GAC, *Reverse*, ATA ATT TAT ATA ATG ATT AAC AAG ATA GAG GTC GAC ACG TCA GAA ATC GAT GAA TTC GAG CTC G. Control primers for the verification of successful recombination: *Forward*, GGA CGA ACT ATA AAC GAG (Prp40 specific bp 322–339); *Reverse*, GTC GAC CTG CAG CGT ACG (pYM8 specific S3R primer [43].

To determine the growth rate of the FF deletion strains cell densities (OD_600_) were normalized and then measured hourly over 540 min.

## Authors' contributions

PU has conceived this study. PU and CE performed yeast two-hybrid screens. CE mapped interaction domains, carried out the pull-downs, the SPOT-synthesis and mapping experiments. CE also made the in vivo FF domain deletions and characterized their phenotype. CE and PU wrote the paper. All authors read and approved the final manuscript.
